# The role of m6A RNA methyltransferase METTL3 in drug resistance mechanisms in acute myeloid leukemia

**DOI:** 10.1007/s44313-026-00123-8

**Published:** 2026-01-30

**Authors:** Suresh Prajapati, Charmi Jyotishi, Mansi Patel, Reeshu Gupta

**Affiliations:** 1 Parul Institute of Applied Sciences, Parul University, Post Limda, Waghodia Road, Vadodara, Gujarat 391760 India; 2 Research and Development Cell, Parul Institute of Applied Sciences, Parul University, Post Limda, Waghodia RoadGujarat, Vadodara, 391760 India

**Keywords:** Acute Myeloid Leukemia, METTL3, Drug resistance, PROTACs

## Abstract

This review examines the role of METTL3, a core RNA methyltransferase, in therapeutic resistance in acute myeloid leukemia (AML) and discusses emerging strategies to address this challenge. METTL3 regulates N^6^-methyladenosine (m^6^A) modifications on transcripts involved in key cellular processes, including apoptosis (BCL2, MCL1), metabolism (PGC-1α, CSRP1), proliferation (MYC), autophagy (FOXO3), and bone marrow microenvironmental interactions (ITGA4, AKT1). These modifications enhance the stability and translation of resistance-associated genes, supporting leukemic cell survival under treatment pressure. Pharmacological targeting of METTL3 has shown efficacy in preclinical AML models. Inhibitors such as STM2457, METTL3-directed PROTACs, and rational drug combinations with agents including venetoclax, anthracyclines, and ATRA, have reversed resistance phenotypes and impaired leukemic cell fitness. Beyond canonical resistance mechanisms, METTL3 also regulates noncoding RNAs, autophagy, and metabolic–epigenetic crosstalk, including histone lactylation, linking epitranscriptomic regulation to broader resistance pathways. By integrating molecular, cellular, and microenvironmental evidence, this review underscores METTL3 as a central driver of drug resistance and a promising therapeutic target in relapsed or refractory AML. Unlike previous summaries, it highlights the convergence of METTL3-mediated m^6^A modifications with noncoding RNA regulation, autophagy, and niche adaptation, and critically evaluates emerging therapeutic approaches, including catalytic inhibitors, PROTACs, and natural compounds.

## Introduction

Acute myeloid leukemia (AML) is a hematological malignancy arising from myeloid progenitor cells or lymphoid-primed multipotent progenitors, characterized by clonal proliferation of immature, undifferentiated myeloid cells. Several treatment approaches, including chemotherapy, hematopoietic stem cell transplantation, immunotherapy, and targeted molecular therapies, enable patients with AML to achieve complete remission. Standard induction therapy for younger patients and medically fit older adults consists of cytarabine (Ara-C) for 7 days combined with an anthracycline antibiotic, such as daunorubicin or idarubicin, for 3 days (“7 + 3”), followed by consolidation chemotherapy or allogeneic stem cell transplantation (SCT). SCT remains the cornerstone of AML treatment and the only potentially curative option. In relapsed or refractory (R/R) disease, salvage chemotherapy regimens, such as fludarabine, cytarabine, and granulocyte colony-stimulating factor (FLAG) and FLAG combined with idarubicin (FLAG-IDA) are commonly used.

First-line therapy for AML leads to complete remission in approximately 60–80% of younger adults and 40–60% of older adults (age > 65 years). Despite initial responses, relapse occurs in nearly 60% of older adult patients, and overall treatment failure exceeds 85% in cases [1]. Drug resistance is a major contributor to these outcomes and markedly limits long-term survival in AML. Resistance arises from multiple molecular mechanisms, including altered drug transport and metabolism, enhanced DNA repair, leukemic stem cell survival, metabolic reprogramming, dysregulated autophagy, apoptosis/necroptosis inhibition, and dynamic interactions within the tumor microenvironment [1, 2]. However, the molecular landscape driving drug resistance in AML remains poorly understood. Clarifying these mechanisms is essential for developing more effective therapies.

Epigenetic and epitranscriptomic modifications have emerged as pivotal regulatory mechanisms in cancer and include DNA methylation, histone modifications, RNA methylation, and noncoding RNAs. Among RNA modifications, N^6^-methyladenosine (m^6^A) is recognized as the most abundant internal modification in messenger RNA (mRNA), and regulates several RNA metabolic processes, such as splicing, stability, nuclear export, and translation [3].

METTL3 is a core component of the m^6^A methyltransferase complex. It drives oncogenic processes, including impaired differentiation, increased proliferation, resistance to cell death, and therapeutic resistance, making it a promising therapeutic target for AML. The METTL3 inhibitor, STM2457, has demonstrated efficacy in preclinical models by suppressing AML cell growth, promoting cellular differentiation, and inducing apoptosis [4].

This review summarizes the structural and biological features of METTL3 in AML, focusing on its emerging role in drug resistance.

## ***METTL3: catalytic architecture and mechanistic role in m***^***6***^***A RNA methylation***

**Fig. 1:**
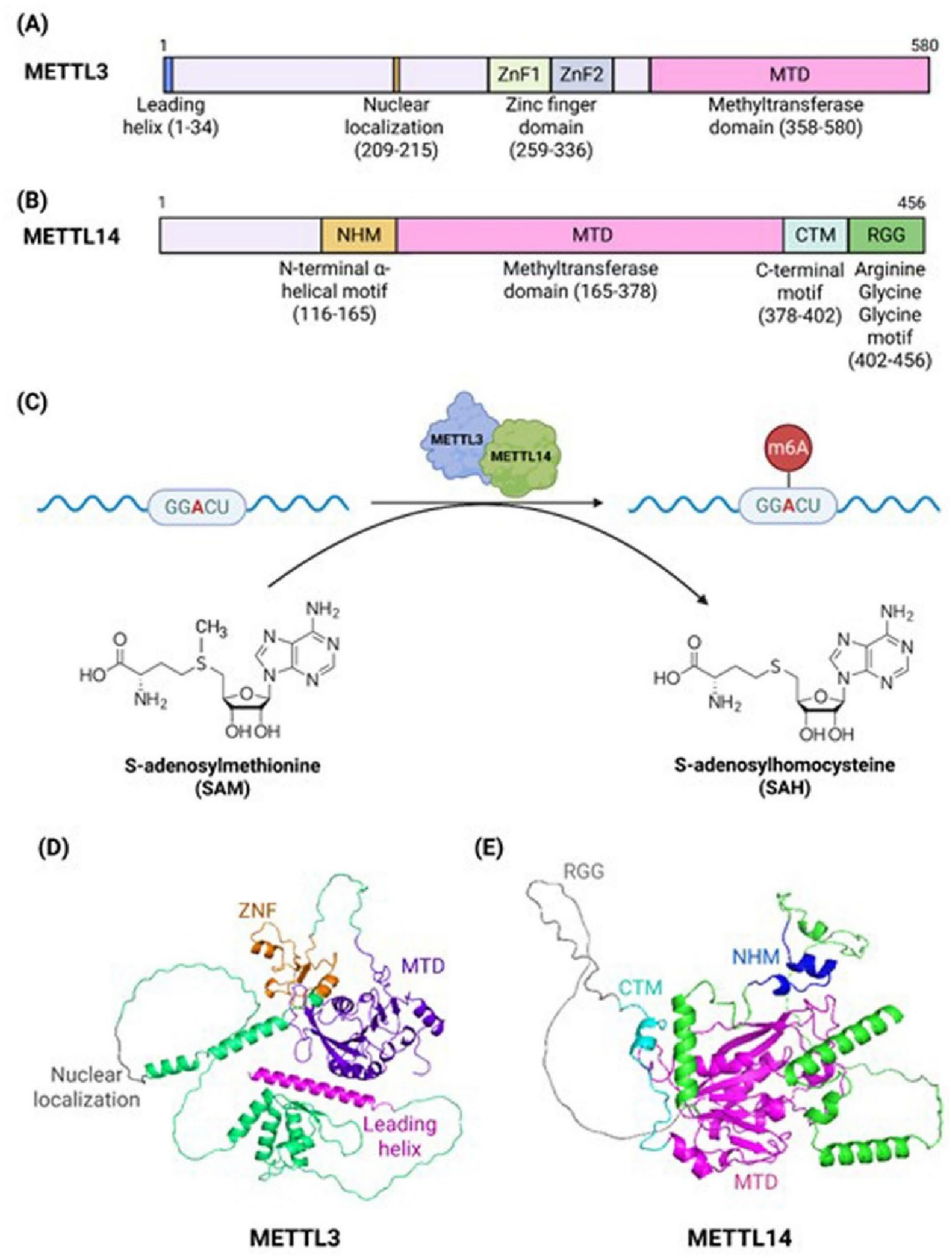
Domain structure of METTL3 and its catalytic role in m^6^A RNA methylation. **A** Schematic representation of METTL3 protein domains, which are responsible for its catalytic activity. **B** Schematic representation of METTL14 protein domains. **C** The METTL3–METTL14 complex catalyzes N-methyladenosine (m^6^A) modification of consensus RRACH motifs (e.g., GGACU) in RNA. The methyl group from the donor S-adenosylmethionine (SAM) is transferred to the adenosine base, producing S-adenosylhomocysteine (SAH) as a by-product, resulting in the formation of m.^6^A-modified RNA. **D** AlphaFold model of METTL3 **E** AlphaFold model of METTL14

METTL3 is a well-conserved 70-kDa enzyme encoded by 11 exons and also referred to as M6A, MT-A70, or SPO8. It consists of a leading helix (1–34), a nuclear localization signal (NLS; 209–215), zinc finger domain (259–336) and a methyltransferase domain (MTD). It serves as the central methyltransferase (“writer”) responsible for installing m^6^A modifications on mRNA (Fig. 1A). This methylation is catalyzed by its S-adenosylmethionine (SAM)-binding domain, primarily in conjunction with METTL14. The protein domains of METTL14 (Fig. 1B) are essential for catalytic activity. METTL14 forms a heterodimeric complex with METTL3, which also associates with additional factors, such as Wilms’ tumor 1–associated protein (WTAP), VIRMA (KIAA1429), CBLL1 (E3 ubiquitin ligase Hakai), RBM15, and ZC3H13. These factors contribute to the complex by recruiting it to the m^6^A consensus RRACH (R = A/G, H = A/C/U) motif of RNA, stabilizing its proper localization [5], promoting preferential methylation at specific transcript sites [6], regulating mRNA splicing and RNA processing, directing the complex to U-rich RNA regions, and guiding it to specific nuclear compartments [7]. METTL16 is an independent m^6^A methyltransferase that specifically methylates the UACAGAGAA sequence motif and regulates the activity of the METTL3/METTL14 complex [7]. The downstream biological effects of m^6^A-modified transcripts are mediated by “reader” proteins that recognize and bind m^6^A marks.

METTL3 and METTL14 form a stable, butterfly-like antiparallel 1:1 heterodimer that significantly enhances methylation efficiency. METTL3 serves as the catalytic subunit, while METTL14 supports RNA target recognition and stabilizes the complex. Mutations in the catalytic site of METTL14 do not affect the methyltransferase activity of the METTL3–METTL14 complex. Structural studies have revealed that SAM and its by-product S-adenosylhomocysteine (SAH) bind within the catalytic pocket of METTL3 but not METTL14, whose closed conformation prevents SAM accommodation (Fig. 1C–E) [8]. In METTL14, steric hindrance between the adenine moiety and the side chains of Trp211^METTL14^ and Pro362^METTL14^ likely blocks SAM from fitting into the active site. In addition, METTL3 contains two CCCH-type zinc finger motifs (ZnF1 [259–298] and ZnF2 [299–336]), which are necessary and sufficient for RNA methylation activity in vitro [5].

The active MTD of METTL3 features a classical α-β-α sandwich fold composed of four α-helices (α1, α2, and α4 on one side; α3 on the other), and eight β-folds (six parallel and two antiparallel), and three 3_10_ helices spanning three gate loops with low sequence similarity: gate loop 1 (residues 396–410), the interface loop (residues 462–479), and gate loop 2 (residues 507–515) [9]. Gate loops 1 and 2 are involved in adenosine recognition, while the interface loop mediates tight binding between METTL3 and METTL14. The MTD of METTL14 (MTD14) spans amino acids 165–378 and is flanked by an N-terminal α-helical region (residues 116–163) and a C-terminal segment (residues 380–402) [10]. Eleven specific residues in METTL3 participate in SAM binding: D377, I378, D395, K513, R536, H538, N539, E532, L533, N549, and Q550. Mutations at D377A, D395A, N539A, and E532A completely abrogated methyltransferase activity, whereas mutations at R536, H538, N549, or Q550 reduce catalytic activity [10]. At the interface between METTL3 and METTL14, a conserved groove facilitates RNA substrate binding during methylation. This groove contains four positively charged amino acids from METTL3 (Arg465, Arg468, His474, and His478) and four from METTL14 (Arg245, Arg249, Arg254, and Arg255). In contrast, similar mutations in METTL14 have little or no impact on catalytic function, reinforcing that METTL3 alone drives catalytic function [10]. However, recent findings show that symmetric demethylation at arginine residues R425 and R445 within the RGG/RG motifs of METTL14, mediated by protein arginine methyltransferases, enhances the catalytic activity of the METTL3–METTL14 complex. This modification promotes m^6^A-dependent gene expression, potentially via the m^6^A reader YTHDF1 in AML cells. Moreover, dual inhibition of PRMT5 and METTL3 using EPZ015666 and STM2457 synergistically suppresses the key m^6^A target genes critical for AML proliferation, suggesting a novel therapeutic strategy for AML treatment [11].

Beyond its methyltransferase function, METTL3 also promotes translation by recruiting the initiation factor eIF3 to a translation initiation complex consisting of CBP80, eIF4E, and the eIF3 subunit eIF3b. For example, METTL3 enhances epidermal growth factor receptor (EGFR) and Hippo pathway effector TAZ protein expression independently of YTHDF1, thereby supporting cancer cell growth, survival, and invasion [12].

## METTL3/METTL14 expression and clinical relevance in AML

**Fig. 2:**
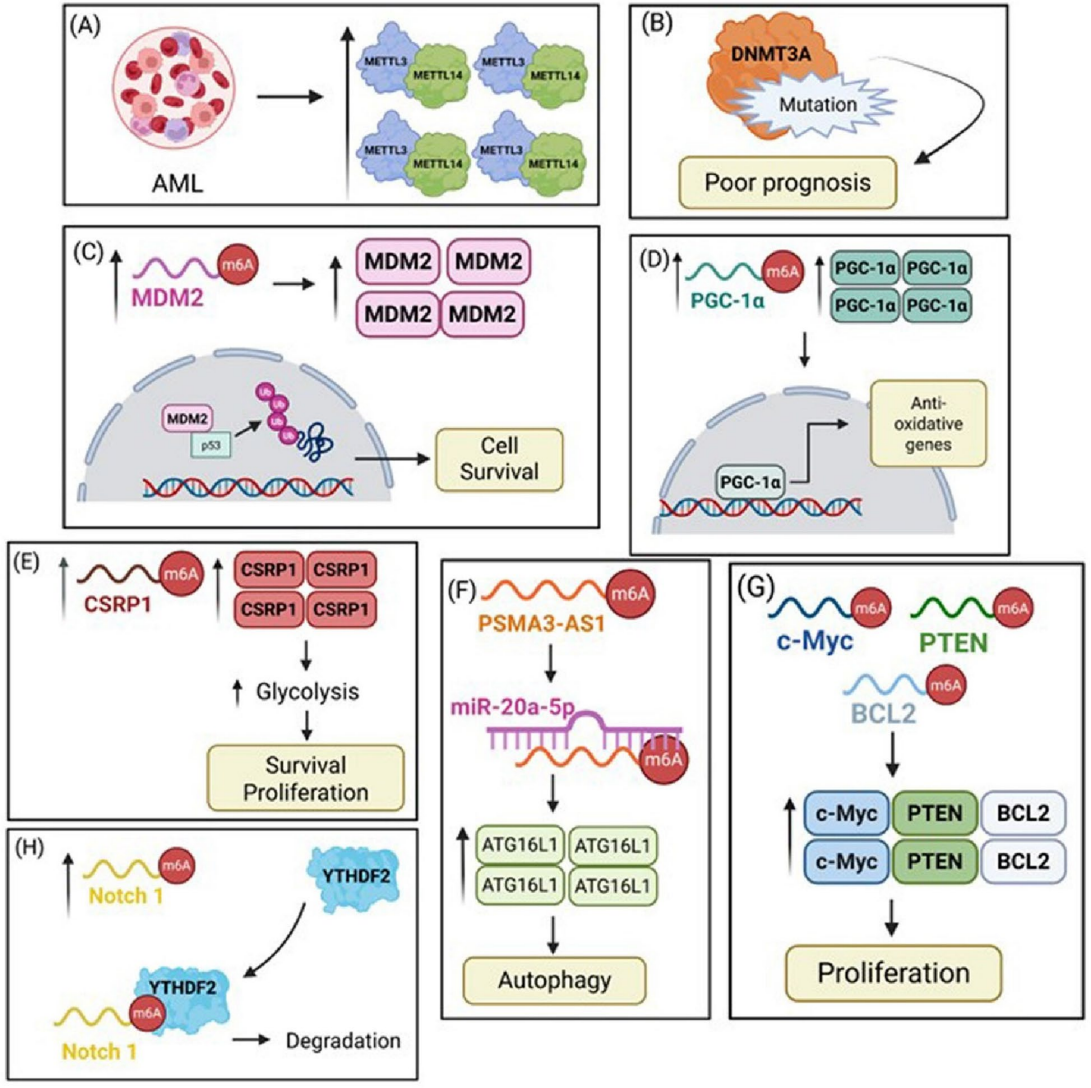
Mechanistic role of METTL3-mediated m^6^A RNA methylation in AML. **A** METTL3 and METTL14 expression is elevated in AML, supporting leukemogenesis through m^6^A-dependent pathways. **B** DNMT3A mutations are linked to poor prognosis in AML, potentially altering methylation patterns and cooperating with m^6^A-modifying enzymes, such as METTL3. **C** m^6^A methylation increased MDM2 expression, promoting p53 degradation via the ubiquitin–proteasome pathway, thereby enhancing leukemic cell survival. **D** METTL3 enhanced PGC-1α expression via m^6^A modification, which promoted the transcription of antioxidant genes, aiding AML cell survival under oxidative stress. **E** m^6^A-modified CSRP1 promoted glycolysis, contributing to increased AML cell survival and proliferation. **F** METTL3-mediated m^6^A modification stabilizes the long noncoding RNA PSMA3-AS1, which sponges miR-20a-5p, leading to upregulation of the autophagy-related gene ATG16L1 and enhanced autophagy in FLT3-ITD⁺ AML. **G** METTL3 promotes the translation of oncogenes, such as *c-MYC, PTEN*, and *BCL2* through m^6^A modification, contributing to leukemic cell proliferation. **H** METTL3 modifies Notch1 mRNA, which is then recognized and degraded by YTHDF2, indicating its regulatory role in suppressing Notch signalling

AML is a prevalent hematologic cancer driven by genetic mutations, transcription factor alterations, and chromosomal abnormalities that disrupt normal gene expression and impair hematopoietic stem cell (HSC) growth and differentiation. METTL3 and METTL14, core components of the m^6^A RNA methyltransferase complex, are frequently overexpressed in AML (except acute promyelocytic leukemia, APL) and correlate with poor patient survival (Fig. 2A). Patients with METTL3-positive AML frequently harbor DNMT3A mutations, possibly due to structural homology between METTL3 and DNA methyltransferases and potential epigenetic crosstalk between DNA and RNA methylation (Fig. 2B). DNMT3A is a well-recognized tumor suppressor in myeloid malignancies, and its mutations are closely associated with impaired differentiation and clonal expansion of HSCs [13]. Coexistence of METTL3 overexpression and DNMT3A mutation supports leukemic stem cell survival by blocking differentiation and preventing normal blood cell formation.

Therapeutically, combining 5-aza-2′-deoxycytidine (5-Aza-Dc) and all-trans retinoic acid (ATRA) suppresses DNMT1 expression, inducing hypomethylation and activating the tumor suppressor miR-34a, which inhibits MYC, leading to cell cycle arrest and apoptosis [14]. Differentiation-inducing agents, such as ATRA, PMA, and 5-Aza-Dc decrease METTL14 expression and reduce overall m^6^A methylation, thereby promoting myeloid differentiation [15–20]. Since METTL3 and METTL14 jointly regulate the stability and translation of key transcripts, such as MYC and MYB, these compounds may therapeutically influence the METTL3–METTL14–m^6^A axis, reducing stabilization of oncogenic transcripts, restoring differentiation, and inhibiting leukemic progression [21].

## Mechanistic roles of METTL3 in leukemogenesis

METTL3 centrally controls RNA methylation in leukemia, influencing cellular events that contribute to malignant transformation. It regulates gene expression in a m^6^A-dependent manner by modulating mRNA stability, translation, and splicing, thereby enhancing leukemic stem cell self-renewal and inhibiting differentiation. Understanding these molecular pathways is essential to understanding the role of METTL3 in leukemogenesis and its potential as a therapeutic target in AML.

### Regulation of p53 signaling and mRNA stability

Knockdown of METTL3 and/or METTL14 significantly decreases m^6^A methylation of MDM2 mRNA, consequently reducing its transcript stability. Since MDM2 negatively regulates p53 by promoting its ubiquitination and degradation, decreased MDM2 levels lead to increased p53 protein. Reactivation of p53 signaling initiates tumor surveillance events, such as cell cycle arrest, apoptosis, and DNA damage repair response, strongly inhibiting AML cell proliferation and shifting the balance from pro-survival to pro-apoptotic states. This underscores the importance of METTL3–METTL14-mediated m^6^A modification in supporting leukemic cell growth by stabilizing oncogenic transcripts, such as MDM2 (Fig. 2C). Targeting this epitranscriptomic axis to reactivate p53-dependent tumor suppression may offer an effective AML treatment approach [20].

### Mitochondrial metabolism and oxidative stress

METTL3 overexpression enhances m^6^A modification of the mitochondrial oxidative stress regulator PGC-1α, stabilizing its transcript and promoting antioxidant defense in leukemic cells (Fig. 2D). METTL3 knockdown in AML cell lines (K562 and MV4-11) led to reduced m^6^A modification of the mitochondrial oxidative stress regulator PGC-1α, and thus decreased YTHDF1-mediated translation. Silencing PGC-1α mimics METTL3 depletion effects by activating MAPK signaling pathway (via increased phosphorylation of P38, c-Jun, and ERK1/2) and suppressing the expression of antioxidant genes (*SOD1, GPX1,* catalase, and *UCP2*). A positive correlation between METTL3 and PGC-1α mRNA levels was observed in primary AML samples and was associated with poor prognosis, suggesting that METTL3-driven metabolic and epigenetic dysregulation jointly promotes leukemogenesis [22].

### Glycolysis and CSRP1 regulation

METTL3 has been shown to promote AML cell survival by modulating metabolic processes. METTL3/YTHDF1 axis also enhances the stability of Cysteine and Glycine-Rich Protein 1 (CSRP1) mRNA via m^6^A modification and prevents its degradation. However, the precise molecular mechanisms underlying this process remain poorly understood. Notably, the expression of CSRP1 is frequently elevated in various cancers, and promotes cell proliferation and survival. This suggests the role of METTL3–YTHDF1–CSRP1 axis in the progression of AML cells by enhancing glycolysis, the main energy source for these cancer cells (Fig. 2E). Interrupting this pathway could offer a potential new direction for targeted therapy [23].

### Upstream transcriptional and epigenetic regulation

Recent findings have identified the transcription factor Yin Yang 1 (YY1) as a key regulator of METTL3 expression. YY1 interacts with histone deacetylases HDAC1 and HDAC3, thereby enhancing METTL3 expression and promoting AML proliferation via liquid–liquid phase separation (LLPS). YY1 is a common zinc-finger transcription factor that binds directly to the METTL3 promoter, which contains an HDAC binding site. Mutation of this site disrupts YY1’s interaction with HDAC1/3, reducing METTL3 expression, and slowing AML progression. The study uncovers a novel regulatory mechanism of METTL3 expression in AML [24].

### Regulation by noncoding RNAs

Circ_0001187, a novel circular RNA functioning as a tumor suppressor in AML, shows reduced expression linked to poor prognosis. It regulates METTL3 stability via a competing endogenous RNA mechanism by sponging miR-499a-5p, which upregulates the E3 ubiquitin ligase RNF113A. RNF113A promotes K48-linked polyubiquitination of METTL3, leading to proteasome-dependent degradation. This degradation reduces m^6^A methylation, destabilizes oncogenic transcripts, suppresses leukemic cell proliferation, and induces apoptosis [25]. Similarly, the long noncoding RNAs, such as PSMA3-AS1, are regulated by METTL3 in FLT3-ITD + AML, which enhances PSMA3-AS1 RNA stability by sponging miR-20a-5p, which in turn regulates the expression of ATG16L1, an autophagy-related gene downregulated in AML. Through the miR-20a-5p/ATG16L1 axis, PSMA3-AS1 promotes autophagy and disease progression in FLT3-ITD + AML cells (Fig. 2F). Another m^6^A-related lncRNA, TRAF3IP2-AS1, shows potential as a prognostic marker and immunotherapy target in patients with AML [26]. Together, these findings highlight a complex network of non-coding RNAs and epigenetic regulators orchestrating METTL3 stability and AML progression [27].

### Control of oncogenic transcripts

Single-nucleotide resolution mapping of m^6^A, coupled with ribosome profiling, revealed that METTL3-mediated m^6^A modification enhanced the translation of *MYC*, *BCL2*, and *PTEN* mRNAs in human myeloid leukemia MOLM-13 cells (Fig. 2G). PTEN, a negative regulator of the PI3K–AKT pathway, contains six m^6^A sites and is a translationally regulated target. Consistent with its role in repressing AKT signaling, the loss of METTL3 led to decreased PTEN translation and a compensatory increase in phosphorylated AKT, which is associated with enhanced differentiation of AML cells. Thus, while METTL3 affects multiple m^6^A-modified targets, the overall outcome favors leukemic proliferation primarily through the upregulation of MYC and BCL2 and altered AKT signaling balance [28]. Additionally, METTL3 cooperates with YTHDF2 to suppress Notch signaling pathway within the hematopoietic system [29] (Fig. 2). METTL3 regulates stem cell fate and is essential for maintaining normal stem cell capacity [30]. These insights establish METTL3 as a pivotal epigenetic regulator and promising therapeutic target for AML (Fig. 2).

## METTL3-mediated mechanisms of drug resistance in AML

Drug resistance in AML is caused by a multifactorial network of molecular alterations. The most commonly recognized mechanisms include: (1) overexpression of drug resistance-related proteins and metabolic enzymes that reduce drug efficacy, such as p-glycoprotein, multidrug resistance protein, glutathione S-transferase, lung resistance protein, topoisomerase II, and protein kinase C; (2) genetic mutations and chromosomal abnormalities that alter drug targets or disrupt apoptosis, including mutations in FLT3, WT1, and the RAS family; (3) dysregulation of microRNAs (miRNAs) involved in drug sensitivity and resistance pathways, such as miRNA-181a (↓ATM), miRNA-638 (↓CDK), miRNA-181a (↓bcl-2) and miRNA-149-5p (reduce apoptosis), and (4) aberrant activation of survival signaling pathways such as PI3K/AKT, MAPK, NF-kB, and autophagy [31]. Recent studies have also identified novel AML-specific resistance mechanisms, highlighting the complexity of treatment failure and the urgent need for targeted therapeutic strategies.

### Venetoclax resistance and the FBXW7/MCL1 axis

**Fig. 3:**
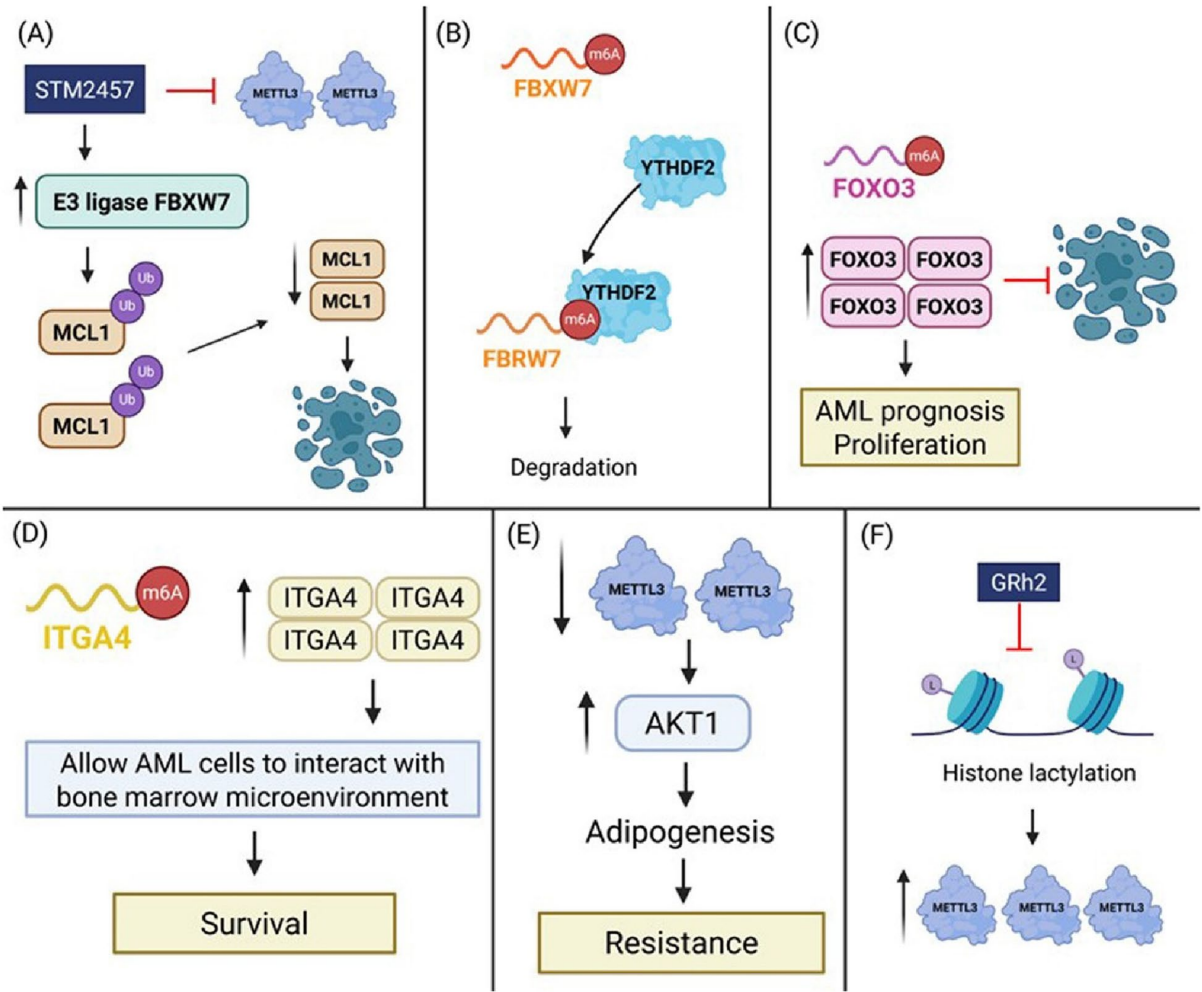
METTL3-mediated m^6^A RNA methylation contributes to drug resistance and leukemic progression in AML. **A** METTL3 inhibition by STM2457 upregulates E3 ubiquitin ligase FBXW7, which promotes MCL1 degradation via the ubiquitin–proteasome pathway, sensitizing AML cells to apoptosis. **B** METTL3-mediated m^6^A modification destabilizes FBXW7 mRNA through recognition by YTHDF2, leading to its degradation and sustained oncogenic signaling. **C** METTL3 amplifies m6A modification and expression of FOXO3, a transcription factor related to unfavourable AML outcome and growth. FOXO3-driven resistance may be suppressed through autophagy inhibition or combination therapy. **D** METTL3 stabilizes ITGA4 mRNA via m^6^A methylation, enhancing AML cell adhesion to the bone marrow microenvironment and promoting cell survival. **E** Reduced METTL3 expression in AML-associated bone marrow stromal cells (BMSCs) increases AKT1 expression, promoting adipogenesis, which contributes to chemoresistance in the leukemia microenvironment. **F** Histone lactylation enhances the expression of METTL3 in APL. A histone deacetylase inhibitor, Ginsenoside Rh2 (GRh2) suppresses lactylation and METTL3, which sensitize cells to drug resistance

The two critical mediators of venetoclax resistance are MCL1 and MYC. METTL3 modified E3 ligase FBXW7 mRNA expression via m^6^A modification and leads to its recognition by the m^6^A reader YTHDF2, ultimately inhibiting the expression of FBXW7. The inhibition of METTL3 by STM2457 prevented this modification. This synergy is partially attributed to STM2457-induced upregulation of the E3 ligase FBXW7, which facilitates MCL1 degradation through the ubiquitin–proteasome pathway. These results suggested that METTL3 inhibitors could reduce venetoclax resistance in AML and, therefore, should be explored along with venetoclax (Fig. 3A and B) [32].

### METTL3 in oncogenic and metabolic regulation

In addition to its role in the venetoclax response, METTL3 stabilizes major oncogenic transcripts that are pertinent to metabolic activities and cell survival. METTL3 elevation in AML facilitates m^6^A methylation of transcripts encoding MYC, BCL2, and PTEN, which increases their ability to be translated and maintains leukemic survival, propagation, and stemness. These findings underscore the broader role of METTL3 in sustaining oncogenic signaling and metabolic stability in AML cells [28].

### METTL3 and anthracycline resistance: the FOXO3–autophagy axis

FOXO3 expression positively correlated with METTL3 expression in anthracycline-resistant AML cells. METTL3 knockdown significantly decreased mRNA stability and levels of FOXO3 protein, supporting the hypothesis that m^6^A modification by METTL3 promotes FOXO3 expression (Fig. 3C). The level of m^6^A-modified FOXO3 mRNA in the resistant AML cells was considerably higher than that in their sensitive counterparts (Fig. 3C). Consistent with these findings, database and Kaplan–Meier survival analyses, along with RT-qPCR validation, revealed that elevated FOXO3 expression was associated with poor clinical prognosis in patients with AML. Functionally, lentiviral overexpression of FOXO3 in AML cells promoted cell proliferation and inhibited apoptosis, further supporting its role as a survival factor for drug resistance. Moreover, inhibition of autophagy using Bafilomycin A1 (Baf.A1), a specific vacuolar H⁺-ATPase inhibitor, significantly enhanced the cytotoxic effect of Adriamycin in both anthracycline-resistant AML cells and FOXO3-overexpressing models (Fig. 3D). These findings indicate that METTL3-driven, FOXO3-mediated autophagy plays a key role in anthracycline resistance in AML [33].

### METTL3 and AML cell homing via ITGA4

METTL3 enhances the homing and engraftment of circulating AML cells by m^6^A-modifying ITGA4 mRNA, which increases ITGA4 protein levels (Fig. 3D). ITGA4 (Integrin alpha-4) is a cell adhesion protein critical for AML cell interactions with the bone marrow microenvironment, contributing to their preservation and survival in protective niches. Interestingly, both the drug-resistant and homing phenotypes were reversed by METTL3 inhibition, indicating a new connection between m^6^A methylation and BM niche interactions, in addition to the promising use of this technique in the treatment of relapsed or refractory AML [34]. Simultaneously, in the bone marrow microenvironment, METTL3 reduces the adipogenesis of bone marrow stromal cells (BMSCs) by regulating AKT protein expression. Downregulation of METTL3 in AML-related BMSCs resulted in elevated levels of AKT with subsequent stimulation of adipogenesis and drug resistance in AML (Fig. 3E). Together, these results highlight the dual role of METTL3 in governing both leukemic cell behaviour and microenvironmental remodeling, offering a potential therapeutic avenue for relapsed or refractory AML through targeted modulation of the m^6^A–AKT–adipogenesis axis [35].

### METTL3 and histone lactylation–driven ATRA resistance

Histone lactylation, distinct from histone acetylation and caused by lactate accumulation, has emerged as a key gene expression regulator. Increased histone lactylation enhances METTL3 expression, promoting ATRA drug resistance in APL. This modification boosts METTL3 activity and stability, leading to sustained m^6^A methylation of downstream transcripts that favor cell survival and ATRA drug resistance. Inhibition of histone lactylation by the natural compound 20(S)-ginsenoside Rh2 (GRh2) suppresses METTL3 expression and enhances ATRA sensitivity by inhibiting m^6^A methylation of key resistance-associated transcripts. This results in increased differentiation and apoptosis in APL cells (Fig. 3F). These findings reveal a mechanistic link between histone lactylation and METTL3-mediated m^6^A modification in the development of ATRA resistance [36].

**Fig. 4:**
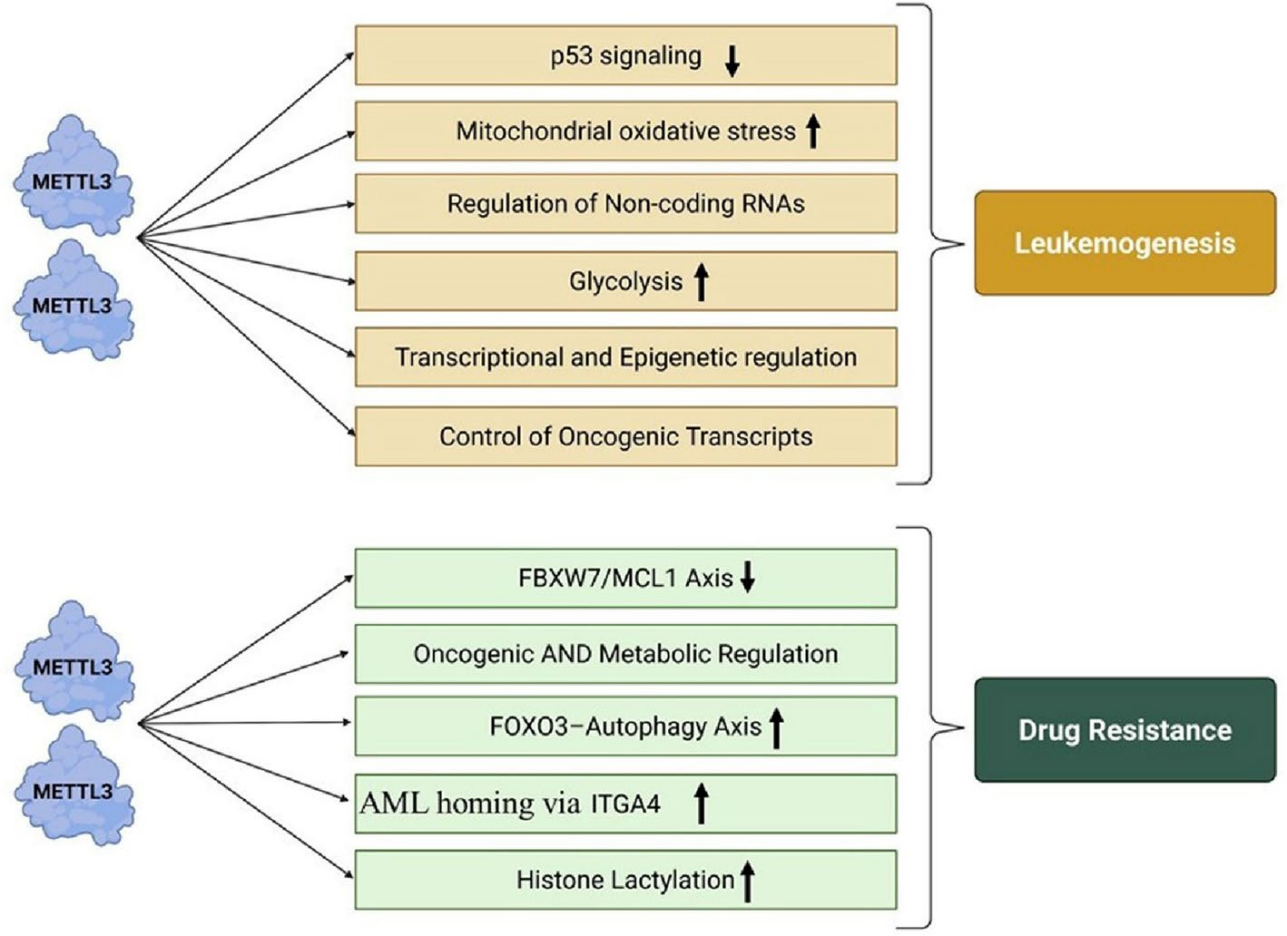
Integrated mechanistic framework showing how METTL3 regulates transcriptional, metabolic, epigenetic, and microenvironmental pathways contributing to leukemogenesis and drug resistance in AML

To provide an integrated overview of METTL3-driven pathways, a unified graphical overview (Fig. 4) summarizes how METTL3 regulates multiple interconnected pathways that promote leukemogenesis and drug resistance in AML.

Across various AML and APL contexts, METTL3 plays a key role in drug resistance by regulating, via m^6^A-based post-transcriptional modification, genes involved in apoptosis, proliferation, autophagy, niche-interaction, and stromal cell behavior. Overcoming resistance and improving outcomes in refractory or relapsed leukemia will potentially require targeting METTL3 with small molecule inhibitors, such as STM2457, natural compounds, including isoliquiritigenin or GRh2, or disrupting downstream effectors. A multipronged approach modulating METTL3-mediated mRNA changes—affecting AML cell-niche communication and adipogenesis of BMSCs—may prove effective in conquering chemoresistance in AML.

### Therapeutic targeting of METTL3 in AML

To date, three mechanistically distinct classes of METTL3 modulators have been identified, namely substrate-competitive inhibitors, allosteric inhibitors, and proteolysis-targeting chimeras (PROTACs) [37]. Among substrate-competitive METTL3 inhibitors, compounds such as UZH2 and those developed by Storm Therapeutics, including STM2457 and EP652, have demonstrated potent antiproliferative activity in AML cell lines. These inhibitors effectively suppress METTL3 catalytic function, highlighting the therapeutic potential of targeting the METTL3–METTL14 complex in myeloid malignancies. Although several of these agents have advanced to preclinical evaluation with encouraging efficacy in AML and other tumor models, all currently remain at the preclinical stage [38]. However, allosteric inhibitors are preferred over substrate-competitive inhibitors because the SAM binding region targeted by substrate-competitive agents is conserved across most methyltransferase enzyme family, raising concerns about nonselective inhibition.

Among the allosteric METTL3 inhibitors, the most well-characterized examples include CDIBA, Eltrombopag, and compound 43n. CDIBA (4-[2-[5-chloro-1-(diphenylmethyl)−2-methyl-1H-indol-3-yl]ethoxy]benzoic acid) was the first reported allosteric inhibitor, binding to a non-conserved pocket within the METTL3–METTL14 complex. Structural optimization of CDIBA improved its binding affinity and selectivity toward METTL3 relative to other methyltransferases. Further optimization led to Eltrombopag and compound 43n, which retain CDIBA’s allosteric binding mode but exhibit enhanced selectivity and reduced off-target activity [39]. Functionally, both Eltrombopag and 43n strongly inhibit METTL3–METTL14 enzymatic activity, leading to decreased global m^6^A methylation and suppressing proliferation of AML cell lines [40, 41]. Table 1 summarizes key METTL3 inhibitors and degraders, including their mechanisms, developers, developmental stages, clinical identifiers, and translational relevance in AML and other malignancies.

**Table 1:** Developmental status of representative METTL3 inhibitors and degraders

Compound	Type/mechanism of action	Selectivity	Developer/institution	Development stage	Clinical trial ID (if available)	Key findings/notes
UZH2	Intracellular METTL3 inhibitor; and blocks m^6^A methyl transfer [42]		University of Zurich	Preclinical	**–**	Demonstrated intracellular inhibition of METTL3 activity and reduced m^6^A levels in AML [43]
STM2457	Binds within the SAM-binding pocket of METTL3 with high affinity and potently inhibited METTL3/14 catalytic activity [44]		Storm Therapeutics Ltd. (UK)	Preclinical	-	Inhibition of METTL3 methyltransferase activity affects m6A modification and translation levels of METTL3-modified mRNAs STM2457 treatment reduces proliferation and colony formation of AML cells, and impairs AML cell engraftment and expansion in human PDX models by inhibiting the self-renewal of leukemic stem cells [4]
UZH1a	Selective and cell-permeable METTL3 inhibitor and shows several favorable interactions of the UZH1a chemical probe with METTL3, some of which are not observed in the METTL3/SAM complex and thus provide selectivity against other SAM-dependent methyltransferases [45]		University of Zurich	Preclinical		Dose-dependent reduction in the m^6^A methylation level of mRNA in several cell lines after 16 h of treatment, which lasted for at least 6 days Importantly, the prolonged incubation did not alter levels of other RNA modifications suggesting selectivity of the developed compound towards other RNA methyltransferases [45]
STC-15	Inhibition of METTL3 stimulates immune cells and activates interferon pathways, leading to the destruction of tumor cells		Storm Therapeutics Ltd. (UK)	Phase 1 dose escalation and cohort expansion study evaluates safety, PK, PD and clinical activity of STC-15 in advanced malignancies	**NCT05584111**	Gene expression pathways related to Interferon (IFN) signalling were enriched in advanced malignancies Tumor regressions were achieved at all dose levels, 60 mg—200 mg three times a week Treatment emergent adverse events, including target-related AE's (e.g., platelet reductions, rash, pruritus) were mainly mild, transient and well managed with supportive care and were not treatment limiting Evidence of M1 macrophages in the TME, consistent with preclinical data Robust METTL3 target engagement indicated by rapid and long-lasting m^6^A inhibition [46]
KH12	PROTAC targeting METTL3 degradation via ubiquitin–proteasome system	Selective METTL3 inhibitor, which targets the SAM binding domain of METTL3 and effectively impedes its methyltransferase activity with a single-digit nanomolar potency [47]	Yonsei University, Korea	Preclinical	**–**	Degrades METTL3 in a dose-, time-, and ubiquitin-dependent manner; reverses differentiation and enhances apoptosis in AML [46]
WD6305	A potent and selective METTL3-METTL14 PROTAC degrader	Selectively targets METTL3-METTL14 complex [48]	Nanjing University of Chinese Medicine, China	Preclinical	**–**	Suppresses m6A modification and the proliferation of AML cells, and promotes apoptosis Affects a variety of signaling pathways related to the development and proliferation of AML [48]
ZW27941	Induces METTL3 degradation via the VHL-mediated proteasomal degradation pathway		University of Florida, Gainesville, FL	Preclinical	**–**	ZW27941 reduces cell viability and degrades METTL3 in a dose-dependent and time-dependent manner Combination of ZW27941 with venetoclax or Ara-C exhibits cooperative antiproliferative activity in AML cell lines [49]
AF151	It forms a stable complex between the E3 ligase von Hippel–Lindau (VHL) and the target-of-interest METTL3, demonstrating efficient METTL3 degradation	METTL3	Johannes Gutenberg-University/Yale University	Preclinical		Showed more pronounced effects on viability inhibition (IC_50_ = 0.45 μM) and more significant m^6^A level reduction in cancer cells than its non-PROTAC parent compounds. By incorporating the indole-nicotinamide chemotype as the METTL3-binding recruiter, this PROTAC is structurally distinct from other METTL3 PROTACs [50]
ZW30441	Cereblon (CRBN)-based PROTAC degraders targeting the METTL3-METTL14 complex	METTL3-METTL14 complex	University of Florida, Gainesville, FL	Preclinical		It showed potent degradation activity against METTL3 and METTL14 with DC_50_ values of 0.44 µM and 0.13 µM, respectively, in the human AML MV4-11 cell line [51]

In addition to specifically designed METTL3 inhibitors, other small molecules have shown METTL3 inhibition and have been tested in cancer treatment. For instance, chidamide induces METTL3 degradation in non-small cell lung cancer [52]. Chidamide is a small-molecule HDAC inhibitor; however, its specific effect on METTL3 degradation in AML remains unexplored. Several clinical trials involving chidamide are currently underway, as summarized in Table 2. Notably, all of these trials evaluate chidamide in combination with standard AML therapies, but none specifically assess its impact on METTL3. Therefore, further research is needed to elucidate potential role of chidamide in modulating METTL3 and its therapeutic implications in AML. Natural compounds have also been explored as potential METTL3 inhibitors. Isoliquiritigenin, a natural flavonoid derived from the root of Glycyrrhiza uralensis (licorice), was identified via molecular docking as a METTL3-binding compound targeting its MTase domain. Treatment with isoliquiritigenin decreased m^6^A methylation in AML cells and exhibited anti-proliferative effects, with a GI50 of 7.43 µM in Molm13 cells and > 10 µM in THP-1 cells [32]. Although isoliquiritigenin shows promising anti-leukemic activity through METTL3 inhibition in preclinical models, no clinical trials currently evaluate its safety or efficacy in humans. Further studies are necessary to assess its pharmacological properties, toxicological profile, and therapeutic potential in AML.

**Table 2:** Chidamide-based clinical trials in acute myeloid leukemia

ID	Purpose	Start date	Completion date	Status	Trial design	Outcome
NCT06066905	To investigate the effect of chidamide and azacitidine on the recurrence rate of MRD-positive AML patients before and after transplantation	10/2023	12/2026	Recruiting	Patients receive chidamide each week for two consecutive weeks, followed by a two-week rest period, completing a 28-day treatment cycle for a total of 12 cycles Azacitidine is administered at 50 mg by subcutaneous injection from days 1 to 5 each week, in 28-day cycles for six cycles The 6-month relapse-free survival (RFS) rate is evaluated to determine treatment efficacy	NA
NCT06386302	To investigate the effect of Chidamide, Venetoclax and Azacitidine in the treatment of newly diagnosed AML, who are not suitable for intensive chemotherapy	08/2024	12/2027	Recruiting	Participants receive a combination of chidamide, venetoclax, and azacitidine (experimental) or venetoclax and azacitidine (comparator). Each treatment cycle lasts 28 days, and participants continue therapy per investigator assessment until disease progression, intolerable toxicity, withdrawal of consent, or fulfilment of other predefined discontinuation criteria The primary efficacy endpoint is composite complete remission (CRc) rate, defined as the sum of complete remission (CR) and complete remission with incomplete hematologic recovery (CRi), after two treatment cycles	NA
NCT05305859	Evaluate the safety and efficacy of Venetoclax, Chidamide and Azacitidine in R/R AML	02/2022	06/2027	Recruiting	Participants receive combination therapy with venetoclax, chidamide, and azacitidine, administered in 28-day cycles for a minimum of two cycles: Each treatment cycle lasts 28 days, and dosing adjustments may be made according to patient tolerance The primary efficacy endpoint is CR rate, defined as < 5% bone marrow blasts by morphology after two months of treatment The overall response rate (ORR) include CR, CRi, and partial remission (PR)	NA
NCT05682755	This Phase I/II clinical trial aims to evaluate the efficacy and safety of chidamide (Epidaza) as maintenance therapy in patients with high-risk acute myeloid leukemia (AML) following allogeneic hematopoietic stem cell transplantation (allo-HSCT)	12/2022	12/2026	Recruiting	Participants receive oral chidamide (Epidaza) beginning after engraftment and continue treatment for up to 180 days post–allo-HSCT, or until disease relapse, unacceptable toxicity, or withdrawal of consent. Primary outcome is to measure progression free survival at the end of two years	NA
NCT06220162	Chidamide in combination with Venetoclax and Azacitidine (VCA) for patients with AML who did not achieve CR/CRi/Morphologic Leukemia-Free State with one cycle of Venetoclax and Azacitidine (VA)	02/2024	12/2026	Recruiting	Enrolled patients receive combination therapy with chidamide, venetoclax, and azacitidine in 28-day treatment cycles. The ORR is calculated as the sun of CR, CRi, morphologic leukemia-free state (MLFS), and PR	NA
NCT05659992	To evaluate the efficacy and safety of venetoclax combined with CACAG regimen (cytarabine, azacitidine, Chidamide aclamycin, granulocyte) in the treatment of newly diagnosed AML	12/2022	08/2024	Recruiting	Patients in this arm receive the CACAG regimen The ORR is defined as the proportion of patients achieving CR, CRi, or PR	NA
NCT05603884	To investigate the effect of VCA regimen followed by Decitabine combined with liposome Mitoxantrone, Cytarabine, and G-CSF (D-MAG) regimen on the treatment of elderly patients with newly diagnosed AML	12/2022	12/2026	Recruiting	Patients receive two cycles of the VCA regimen (venetoclax, chidamide, and azacitidine), followed by two cycles of the D-MAG regimen (decitabine, mitoxantrone, cytarabine, and G-CSF) This four-cycle sequence is subsequently repeated once for a total of eight treatment cycles The primary endpoint is the CR rate	NA
NCT06928376	To compare the efficacy and safety of venetoclax combined with CACAG (cytarabine, azacitidine, chidamide, aclacinomycin, and G-CSF) regimen with the traditional "3 + 7" regimen in the treatment of newly diagnosed intermediate- or high-risk AML	04/2024	10/2026	Recruiting	The CACAG + Venetoclax regimen is administered over one week, with each treatment cycle repeated every 4 weeks for a total of one course The primary efficacy endpoint is the composite complete remission (CRc) rate after one course of treatment, defined as the sum of CR and complete CRi	NA
NCT06532552	Comparison of VA (Venetoclax, Azacitidine), VACl (VA, Cladribine), VACh (VA, Chidamide), and alternating VACl/​VACh in newly diagnosed AML	07/2024	08/2028	Recruiting	Patients receive two cycles of treatment in each of the four groups. Event-Free Survival (EFS) and Cumulative Incidence of Relapse will be measured after 1 year	NA
NCT06068621	To compare the efficacy and safety of venetoclax combined with CACAG regimen with the traditional "3 + 7" regimen in the treatment of newly diagnosed AML	08/2023	01/2026	Recruiting	Patients receive Azacitidine (days 1–7), Cytarabine (days 1–5), Aclacinomycin (days 1, 3, and 5), Chidamide (days 1, 4, 8, and 11), Venetoclax (days 1–14; dose reduced when combined with azole antifungals), and Granulocyte colony-stimulating factor starting from day 0 until agranulocytosis recovery. The primary efficacy endpoint is the ORR after one course of treatment, defined as the proportion of patients achieving CR, CRi, or PR	NA
NCT06084819	To compare the efficacy and safety of venetoclax combined with the CACAG regimen with Best-Available Therapy (BAT) regimen in the treatment of relapsed/refractory AML	08/2023	01/2026	Recruiting	Patients receive treatment as per the standard schedule. The primary efficacy endpoint is the ORR after one course of treatment, defined as the proportion of patients achieving CR, CRi, or PR	NA
NCT03985007	To investigate the therapeutic efficacy and side effects of chidamide, decitabine combined with priming IAG regimen (cytarabine, idarubicin, and concurrent G-CSF) for relapsed or refractory AML (Phase II)	01/2018	03/2021	Completed	The therapeutic regimen comprised chidamide (30 mg orally twice every week for 2 weeks on days 1, 4, 8, and 11), decitabine [20 mg/m2 intravenously daily for 5 days (d1-d5)], and the IAG regimen [cytarabine (10 mg/m2 subcutaneously every 12 h. on days 4–17), idarubicin (5 mg intravenously every other day on days 4, 6, 8, 10, 12, and 14), and concurrent G-CSF (200 μg/m2/day subcutaneously daily on days 3–17)]. The primary endpoint was the remission rate, defined as the proportion of patients achieving CR or CRi after one to two induction courses of the CDIAG regimen, assessed at 1 month	The double epigenetic priming regimen (CDIAG regimen) showed considerably good antileukemia activity in these patients. Adverse events were acceptable according to previous experience [53]
NCT05659992	To evaluate the efficacy and safety of venetoclax combined with CACAG regimen in the treatment of newly diagnosed AML (Phase II)	12/2022	08/2024	Completed	Patients received induction treatment with aclarubicin (10 mg/m2/d on days 1, 3, and 5), azacitidine (75 mg/m2 on days 1–7), cytarabine (75 mg/m2 bid on days 1–5), chidamide (30 mg, twice/week for 2 weeks), and venetoclax (100 mg on day 1, 200 mg on day 2, 400 mg on days 3–14). Granulocyte colony-stimulating factor 5 μg/kg/day was administered. The primary efficacy endpoint was the ORR, defined as the percentage of patients achieving a CR, CRi, or PR after one course of treatment	After one CACAG-VEN cycle, the overall response rate was 96.7%, with a CRc of 93.3% (including 86.7% in adverse-risk patients). Two treatment cycles achieved a 100% CRc rate. The 12-month overall survival was 69.7%. Median recovery times after induction were 19 days for platelets ≥ 50 × 10^9^/L and 17 days for ANC ≥ 0.5 × 10^9^/L. Single-cell RNA sequencing indicated minimal change in immune cell composition after tumor cell clearance [54]

Despite growing interest in METTL3 as a therapeutic target, several challenges hinder the clinical translation of METTL3 inhibitors. First, METTL3 regulates erythropoiesis; therefore, its inhibition may induce severe anemia, as demonstrated by decreased red blood cell counts, hemoglobin, and hematocrit in *in-*vivo model [55]. Second, METTL3 inhibitors may interfere with the differentiation and development of normal tissues [56]. Third, most current inhibitors exhibit off-target liability, as many inhibitors target the conserved SAM-binding pocket, raising concerns about cross-reactivity with other RNA, DNA, and protein methyltransferases [4]. Fourth, the role of METTL3 inhibitors is highly context-dependent, exerting either oncogenic or tumor-suppressive effects based on cancer type and microenvironment [57], complicating the prediction of systemic safety and dosing strategies. Additionally, limited pharmacokinetic and pharmacodynamic data, including poor bioavailability and solubility, and low stability have slowed preclinical progression [58]. Another major hurdle is the lack of reliable biomarkers to identify responsive patients [42]. Finally, the possibility of adaptive resistance mechanisms, including compensatory upregulation of other m^6^A regulators (METTL14, WTAP, FTO), must be considered in clinical design [59]. Addressing these challenges is essential for translating METTL3 inhibition from preclinical promise to viable AML therapy.

In contrast, Proteolysis Targeting Chimera (PROTACs) offer a valid alternative to small-molecule inhibitors. Conventional small-molecule inhibitors act in an occupancy-dependent manner, requiring sufficient occupancy of the protein’s active site to inhibit function. Because these interactions tend to be reversible, high drug concentrations are often needed to maintain target occupancy. PROTACs, however, operate via an event-driven process: a PROTAC molecule connects the target protein to an E3 ligase, inducing ubiquitination and proteasomal degradation of the target. After degradation, the PROTAC is released intact and can repeat this catalytic cycle. This catalytic and recyclable mechanism minimizes the need for large drug doses and avoids some limitations associated with traditional inhibitors [60, 61]. By targeting the entire protein for degradation, PROTACs eliminate both enzymatic activity and scaffolding roles, effectively functioning as a chemical knockout of the protein. Unlike traditional inhibitors, which transiently inhibit METTL3 activity, PROTACs irreversibly remove the protein from the cell, blocking all its functions. Due to their high specificity, tissue penetration, and molecular stability, PROTACs provide long-lasting therapeutic effects. Another major benefit is their ability to target intracellular and mutated proteins often inaccessible to conventional inhibitors. Although low oral bioavailability remains a limitation, ongoing chemical optimization and formulation strategies are improving this aspect. In summary, PROTACs represent a novel and powerful approach for selectively degrading disease-related proteins, offering greater precision and sustained efficacy compared to conventional small-molecule inhibitors [62]. STC-15 represents the first-in-class METTL3 degrader to enter human clinical evaluation, demonstrating the translational feasibility of RNA methyltransferase targeting via protein degradation (Table 3). Other METTL3-directed PROTACs, including KH12, WD6305, and ZW27941, remain in preclinical development. These have demonstrated potent and selective METTL3 degradation in AML cell lines such as MOLM-13, MV4-11, and NB4 [47, 48, 63]. Additionally, KH12 effectively reverses differentiation blockades and exhibits superior anti-proliferative effects compared to traditional small-molecule inhibitors [47]. A recent study designed several PROTAC molecules targeting METTL3, identified as compounds 14, 20, 22, 24, and 30. These compounds vary in linker length, chemical composition, and structural flexibility. The most efficient PROTACs had shorter linkers (three to five methylene units) and incorporated hydrophobic moieties, such as piperidine, piperazine, or triazole. Among these, compounds 20 and 22 reduced METTL3 levels by over 50% in AML cell lines, such as MOLM-13, NOMO-1, THP-1, and Kasumi-1 [42]. This study highlights the importance of optimizing linker length and composition to enhance METTL3 degradation and antileukemic activity [64].

**Table 3:** Clinical trials investigating STC-15 (METTL3 Inhibitor) in advanced malignancies

ID	Purpose	Start date	Completion date	Status	Trial design	Outcome
NCT06975293	To investigate the safety, tolerability, and antitumor activity of STC-15 in combination with toripalimab in locally advanced unresectable or metastatic tumors	05/2025	01/2026	Recruiting	This study comprises two parts: a combination dose escalation part (Phase 1b) followed by an assessment of the combination treatment's antitumor activity (Phase 2). This study involves adult participants with advanced malignancies to characterize the safety, tolerability, PK, and clinical activity of STC-15 in combination with toripalimab	NA
NCT05584111	To evaluate the safety, pharmacokinetics, pharmacodynamics, and clinical activity of STC-15 in subjects with advanced malignancies	11/2022	12/2024	Completed	This Phase 1, multi-center, open-label, first-in-human study evaluates multiple ascending daily oral doses of STC-15 in Q3W treatment cycles in a 3 + 3 cohort design with dose levels determined by a modified Fibonacci algorithm. The study was designed to systematically assess safety and tolerability, pharmacokinetics, pharmacodynamics, and clinical activity of STC-15 in adult subjects with advanced malignancies	Tumor regression was observed across all tested dose levels (60–200 mg, thrice weekly). Treatment-emergent adverse events were transient, manageable with supportive care, and not dose-limiting [46]

Although PROTAC-mediated degradation holds promise for targeting previously undruggable proteins, several mechanistic and translational challenges remain. One key issue is distinguishing between “molecular glue” mechanisms and PROTAC-mediated degradation. In addition, the catalytic rather than stoichiometric nature of PROTACs complicates conventional pharmacokinetics and pharmacodynamic assessments, underscoring the need for specific PK/PD evaluation systems tailored to PROTACs. Other limitations include the requirement for high-affinity ligands to target proteins, especially where PPI occur, and the limited repertoire of E3 ligases currently exploited—mainly VHL, CRBN, cIAPs, and MDM2—out of the over 600 E3 ligases encoded in the human genome. Maximizing degradation efficiency, selectivity, and minimizing off-target effects in diverse biological contexts remain key objectives in the field [65].

### Future perspectives and research directions

Although substantial progress has been made in elucidating the role of METTL3 in AML pathogenesis and resistance to therapy, several areas remain underexplored and warrant further investigation. A deeper understanding of the interplay between METTL3-mediated m^6^A modification and other epigenetic or genetic mechanisms could reveal novel regulatory networks driving resistance. Specifically, the integration of METTL3 with chromatin remodeling, noncoding RNAs, and transcriptional regulators in the context of therapeutic failure remains unclear. The development of predictive biomarkers for METTL3-targeted therapies is another important research direction. Stratifying patients based on METTL3 expression levels, m^6^A modification signatures, or downstream effectors such as MYC, MCL1, or FOXO3 could enhance therapeutic precision and help identify individuals most likely to benefit from METTL3 inhibition. Such efforts may benefit from the incorporation of high-throughput transcriptomic and proteomic profiling technologies.

Furthermore, the long-term effects of METTL3 inhibition on normal hematopoiesis are not yet fully understood. Since METTL3 also plays a role in regulating normal hematopoietic stem cell self-renewal and differentiation, prolonged or systemic inhibition may pose a risk to bone marrow function. Future studies should evaluate potential hematologic toxicities in preclinical models and clinical settings.

The emergence of resistance to METTL3-targeting agents presents a potential challenge. As with other targeted therapies, compensatory mechanisms or alterations in m^6^A machinery—such as upregulation of METTL14 or alternative reader proteins—could diminish therapeutic efficacy over time. Addressing this issue may require combined approaches that co-target downstream pathways or parallel resistance mechanisms. Additionally, the application of single-cell and spatial multiomics approaches could provide unprecedented insights into METTL3's role in intratumoral heterogeneity and therapeutic responses. By capturing dynamic changes in m^6^A methylation, gene expression, chromatin accessibility, and metabolic states at the single-cell level, these technologies may help uncover lineage-specific vulnerabilities and resistance niches within the AML microenvironment. Finally, the role of METTL3 in modulating immune responses and inflammatory signaling in AML remains largely unexplored. Given the increasing interest in immunotherapeutic strategies for AML, understanding whether METTL3 influences immune evasion, antigen presentation, and cytokine signaling could open new avenues for the development of combination regimens involving METTL3 inhibitors and immune-based therapies.

In conclusion, targeting METTL3 represents a promising strategy to overcome drug resistance in AML; however, its full clinical potential will depend on resolving key biological questions, optimizing patient selection, and anticipating resistance pathways. Multidisciplinary approaches integrating molecular biology, bioinformatics, and translational research are critical for advancing METTL3-based therapies from the bench to the bedside.

**Table:** 

Clinical Implications
Increased METTL3 expression and distinct m^6^A methylation profiles may serve as valuable biomarkers to identify patients who are most likely to benefit from METTL3-targeted or combination therapies
Since METTL3 also supports normal hematopoietic stem cell function, complete inhibition could temporarily disrupt blood cell formation. Therefore, optimizing dosing and employing selective inhibition strategies are essential to minimize potential hematologic toxicity
Preclinical studies with METTL3 inhibitors, such as STM2457, STC-15, and UZH2, as well as METTL3 degraders, such as KH12 and WD6305, demonstrate that these agents can enhance the effectiveness of standard AML treatments
Combining METTL3-targeted drugs with BCL2 inhibitors (e.g., venetoclax) or epigenetic agents (e.g., azacitidine, decitabine) shows promise and warrants further clinical investigation in patients with refractory AML
METTL3 inhibition sensitizes AML cells to venetoclax, anthracyclines, and hypomethylating agents by promoting apoptotic reprogramming and overcoming drug resistance. Thus, combining METTL3 degraders or inhibitors with BCL2 inhibitors (venetoclax) or epigenetic modulators (azacitidine, decitabine) represents a rational and promising therapeutic strategy that merits clinical evaluation

## Data Availability

No datasets were generated or analysed during the current study.
